# Immunological Modulation in Long-Term Karate Practitioners

**DOI:** 10.1155/2018/1654148

**Published:** 2018-06-26

**Authors:** Juan M. Manzaneque, Francisca M. Vera, Gabriel A. Carranque, Francisco M. Rodríguez-Peña, Federico Navajas, María J. Blanca

**Affiliations:** ^1^Departamento de Psicobiología y Metodología, Universidad de Málaga, Andalucía Tech, Málaga, Spain; ^2^Departamento de Cirugía, Universidad de Málaga, Andalucía Tech, Málaga, Spain; ^3^Unidad de Gestión Clínica de Laboratorio, AGS Este de Málaga-Axarquía, Málaga, Spain

## Abstract

Karate is a Japanese martial arts system with potential physical and psychological benefits. However, karate has been scarcely investigated from a psychobiological perspective, and its effects on the immune system remain virtually unknown. We designed the present study with the aim of analyzing the effects of karate practice on immunological parameters. 27 healthy male volunteer subjects participated in the study, 15 in the experimental group and 12 in the control. Experimental subjects were all karate players who had practiced this martial art for a minimum of three years attending regular lessons at a karate training center, in the evening, two to three days per week. Blood samples for the quantification of immunological parameters (total leukocytes, neutrophils, monocytes, eosinophils, basophils, lymphocytes, IgG, IgA, and IgM) were taken in both groups. A t-test for independent groups was performed in each dependent variable; a value of p<0.05 was considered to be significant. Karate practitioners exhibited a significantly higher number of total leukocytes (p<0.02), monocytes (p<0.01), and lymphocytes (p<0.01), a higher percentage of monocytes (p<0.01), and greater serum concentrations of IgG (p<0.02) and IgM (p<0.01). Our findings show that long-term karate practice is related to a broad modulation of immune parameters, including total and specific leukocyte counts, as well as immunoglobulin concentrations. This peculiar immunomodulatory profile, apart from its psychobiological relevance, may have noteworthy clinical implications.

## 1. Introduction

Karate is a Japanese martial art [1] which is currently one of the most popular forms of martial arts worldwide [2, 3]. It is practiced both as a form of self-defense as well as a discipline with potential physical and psychological benefits [4].

There are currently over a couple of hundred of scientific studies on the issue of karate. The majority of these studies have focused on the effects that specific interventions are exerted on karate performance [3, 5–7], mainly on elite athletes [8–10], on the physiology of karate [11–20], on the physical dynamics of karate per se [21–27], and on injuries derived from karate practice [28–36].

Karate has been reported to produce physiological and/or psychological gain [1, 37–42], improve visual perception [43] and body balance [44], and increase strength and flexibility [45], among other effects. Likewise, karate has been found to be effective in helping subjects with very different conditions ranging between disruptive behavior [46], blindness [47], autism [48], and even spinal cord injury damage [49].

From a psychobiological perspective, albeit there are several studies in relation to karate, the number is still limited [6, 14, 38, 40, 41, 50–53] and very little is actually known with respect to the long-term psychobiological effects associated with karate practice. In particular, although physical exercise has been consistently reported to be associated with immune changes [54–56], the possible effects of karate over the immune system remain virtually unknown. Therefore, given this striking lack of data, the present research was designed with the aim of shedding light on the effects that long-term karate practice can exert over immune parameters.

## 2. Material and Methods

### 2.1. Participants

27 male participants, with ages ranging from 20 to 50, participated in this study. The experimental or karate group consisted of 15 long-term karate practitioners (12 with black belt and 3 with brown belt), while the control group was made up by 12 ordinary subjects with no previous experience of karate or other martial arts. The main inclusion criterion for both the experimental and control groups was being in good health and following regular lifestyle habits. Potential participants were excluded if they had any diagnosed health condition and/or had taken prescribed medication during the three months prior to the study period. The experimental subjects were recruited from several karate training centers in the city of Malaga throughout a period of three weeks. The control group was selected from within a group of healthy subjects without disease or suspicion of disease that were programmed to go to hospital for a routine medical check-up. The reason for these individuals to be in hospital, therefore, was not owing to any pathology that could have compromised the representativity of the control group. These subjects had the same characteristics (age, height, weight, and trait anxiety) as those of the experimental group (Table 1) but had no previous experience with karate or any other martial art and did not practice sports regularly. The study protocol was conducted according to the Declaration of Helsinki and was approved by the Institutional Ethical Committee. Informed consent was obtained from all individual participants.

### 2.2. Intervention

Karate training in the experimental group typically entailed two to three lessons a week, with a duration of about one hour each. Experimental subjects had been following this routine for a minimum of 3 years. Karate practice, which is normally preceded by a brief heating, traditionally consists of “kata” (i.e., a prearranged solo fighting sequence), combat drills (i.e., short fighting techniques practiced with a partner), and “kumite” (i.e., a sparring practice involving two partners). The main feature of traditional karate lies on the balance, in terms of importance and dedication, given to these three components. At initial stages, however, practitioners concentrate more on “kata” and combat drills in order to acquire basic skills that can be later used in “kumite.”

### 2.3. Sampling

Blood samples were taken from all subjects for the quantification of the white blood cell count (total leukocytes, neutrophils, monocytes, eosinophils, basophils, and lymphocytes), as well as the concentrations of immunoglobulins (IgG, IgM, and IgA). Experimental subjects gathered at the hospital (“Unidad de Gestión Clínica de Laboratorio” of AGS Este de Málaga-Axarquía) for a period of one week, coinciding with the appointment of control subjects. Blood was drawn by venepuncture at 9:00 h., centrifuged immediately in a refrigerated centrifuge, and stored at –20°C. For the leukocyte count, 5 ml of blood was introduced into an EDTA tube and analyzed by flow cytometric analysis in an XE-2100 ANALYZER (Roche Diagnostic). The concentrations of immunoglobulins were assessed with a competitive chemiluminescence enzyme immunoassay. All samples were quantified by using MODULAR ANALYTICS E170 (Roche Diagnostics).

### 2.4. Statistical Analyses

An independent sample t-test was performed for each immunological parameter in order to analyze the differences between the means of the control and karate groups. Cohen's d was calculated as an effect size measure [57]. IBM SPSS was used for the statistical analysis, and Shapiro–Wilk test was used for examining the normality assumption, all variable being normally distributed except eosinophils and basophils. In these two cases, nonparametric test such as Mann–Whitney* U* test provided the same results as t-tests. A value of p<0.05 was considered to be significant.

## 3. Results

Results from independent sample t-test are presented in Table 2. Differences were found in total leukocytes, monocytes, monocytes %, lymphocytes, IgG, and IgM, with these parameters being higher in the karate group. According to Cohen's criterion [57], the effect size values indicated a large difference between groups in all significant variables.

## 4. Discussion

The main finding of the present research is that long-term karate practice is associated with a significant immunomodulatory action consisting of an increased number and/or percentage of some leukocytes, specifically monocytes and lymphocytes, as well as higher concentrations of IgG and IgM. These results are, to the best of our knowledge, the first to be published to date associated with karate practice.

The larger number of total leukocytes found in the group of karate practitioners is indeed a most novel result from our research. As a matter of fact, up to now, there seems to be only one study having researched the influence of karate on immune parameters [58]. Nonetheless, while this research measured immunological parameters in elite karate athletes, no significant differences were reported in the leukocyte count. This differs from results found in our study. This difference may be due, at least in part, to the fact that our subjects were regular but ordinary practitioners and not elite karate professionals. Thus, it is likely that the life style derived from elite sports practice, with their typical load of training, may have influenced the immune system, as well as other biological systems, in a distinct manner.

A peculiar finding of our investigation that has not been reported to date in relation to karate practice is the greater number and percentage of monocytes found in the karate group. In this sense, since these changes in monocytes include not only the number but also the percentage of this type of leukocyte, it is important to point out that these monocyte changes may have influenced the elevation of total leukocyte count of our study in a specially significant manner. Nonetheless, the remarkable lack of data on the influence of karate on monocyte count and generally on the total leukocyte count makes it difficult for us to make direct comparisons with other investigations, except with the aforementioned work of Dopsaj et al. [58]. However, Judo, another Japanese martial art, has been studied recently in relation to monocyte count by Shimizu et al. [59], who reported decreases of monocytes in male judo athletes. While this result also differs from ours, it is necessary to emphasize that judo is a very different martial art to karate, as the former relies mainly on grappling techniques, while the latter is based on strikes. In addition, the judo sample of this study by Shimizu et al. [59] consisted of elite Japanese judo athletes, who were actually following weight loss programs preceding completion, and these factors may have indeed played a role in the difference of results. Another work by Shimizu et al. [60], nonetheless, coincides with our results, given that an elevation in the number of monocytes, related to exercise training, was reported.

The larger number of lymphocytes associated with the karate group is also an interesting result from our research. While karate has never been reported to be associated with changes of lymphocyte counts, training in Taekwondo, a Korean martial art similar to karate, has been said to be related to elevations of specific lymphocyte subpopulations [61]. Likewise, a recent investigation of immunoendocrine changes following Marine Corps martial arts training revealed that this program also induced an elevation of lymphocytes [62]. The changes of lymphocyte count induced by martial arts training are a remarkable effect that confirms martial arts can exert a noteworthy action on the immune system reaching even cell components of the adaptive immune response.

Karate practitioners in our study also exhibited a greater concentration of IgA and IgM than control. Albeit there is no specific data available in scientific literature with respect to the effects of karate on immunoglobulin levels, a recent report showed that the practice of jiu jitsu, another form of Japanese martial art and modern combat sport, did not alter the levels of immunoglobulins [63]. Nonetheless, judo, a very similar art to jiu jitsu, from which the former is derived, has been found to decrease the concentrations of immunoglobulins [64]. At any rate, aside from the fact that judo and karate are very different martial arts systems, it must also be pointed out that subjects in this judo study were, once again, professional athletes that had actually been submitted to a weight reduction program; therefore, it is quite likely that these factors may account for the discrepancies between our results and those of this study. It is interesting to note that the higher levels of immunoglobulins found in our research seem to be quite coherent with the rest of the peculiar immune action displayed by the karate group. In this regard, the karate practitioners in our sample exhibited increased values of all of the immune parameters measured where significance was reached. While it is certainly tempting to speculate on the clinical implications of this consistent immune profile, it is indeed beyond the scope of this paper to clarify the clinical relevance that could derive from these results.

The small number of subjects in the sample as well as measuring immune parameters at one time-point only throughout the study may be considered, to a certain extent, a limitation of its overall results. Nonetheless, it is necessary to emphasize that the recruitment of a sample of long-time karate practitioners, who trained on a regular basis for several years without significant gaps, was a difficult task in this investigation. Also, the long-term nature of this practice made it difficult to obtain more than one measure whose evolution could be studied over time. At any rate, future studies should certainly include a larger sample size, more than one measure throughout time, and maybe even several measures per day. This would indeed provide a broader and more consistent perspective of the long-time effects of karate on immune parameters.

## 5. Conclusions

Our findings reveal that long-term karate training is associated with a significant and broad immunomodulatory action which includes cell components from both the innate and the adaptive immune response, as well as the levels of immunoglobulins. This peculiar immunological profile displayed by long-time karate practitioners is a most novel result which lends itself to relevant clinical considerations. We conclude that further research should be conducted to confirm the interesting influence karate appears to exert over the immune system and to shed light on its possible clinical implications.

## Figures and Tables

**Table 1:** Mean and standard deviation (in parenthesis) of age, height, weight, and STAI-R score in the control and karate groups, as well as independent sample t-test and p value.

Variable	Control Group	Karate Group	t	p
Age (yr)	28.75 (9.93)	35.00 (14.05)	-1.30	.20
Height (cm)	180.62 (5.55)	175.86 (8.38)	1.43	.17
Weight (kg)	80.75 (8.72)	78.13 (11.89)	0.54	.30
STAI-R (score)	8.00 (6.24)	12.46 (10.92)	-1.16	.14

STAI-R: Trait Anxiety scale of the STAI.

**Table 2:** Mean and standard deviation (in parenthesis) of immunological parameters, independent sample t-test, one-tailed p value, and Cohen's d.

Variables	Control Group	Karate Group	t	p	d
Leukocytes (x10^9^/L)	5.65 (0.94)	6.87 (1.74)	-2.17	.02	0.87
Neutrophils (x10^9^/L)	3.00 (0.72)	3.45 (1.10)	-1.21	.12	0.48
Monocytes (x10^9^/L)	0.46 (0.19)	0.61 (0.16)	-2.33	.01	0.85
Eosinophils (x10^9^/L)	0.28 (0.17)	0.24 (0.17)	0.60	.27	0.23
Basophils (x10^9^/L)	0.040 (0.029)	0.033 (0.017)	0.80	.22	0.29
Lymphocytes (x10^9^/L)	1.83 (0.37)	2.31 (0.56)	-2.57	<.01	1.01
Neutrophils %	52.88 (7.51)	52.41 (8.56)	0.15	.45	0.06
Monocytes %	7.43 (2.02)	9.04 (1.51)	-2.32	.01	0.90
Eosinophils %	4.32 (2.14)	3.71 (2.78)	0.61	.27	0.24
Basophils %	0.69 (0.47)	0.50 (0.27)	1.20	.12	0.49
Lymphocytes%	32.64 (5.49)	34.35 (7.10)	-0.68	.25	0.27
IgG (mg/dL)	1025.83 (197.08)	1166.71 (109.84)	-2.20	.02	0.88
IgA (mg/dL)	229.33 (76.36)	218.66 (83.16)	0.34	.36	0.13
IgM (mg/dL)	82.82 (23.28)	111.07 (33.63)	-2.37	.01	0.98
